# Peripheral and tumor immune correlates in patients with advanced melanoma treated with nivolumab (anti-PD-1, BMS-936558, ONO-4538) monotherapy or in combination with ipilimumab

**DOI:** 10.1186/1479-5876-12-S1-O8

**Published:** 2014-05-06

**Authors:** Michael A Postow, Diana M Cardona, Janis M Taube, Robert A Anders, Clive R Taylor, Jedd D Wolchok, Margaret K Callahan, Michael A Curran, Alexander M Lesokhin, Joseph F Grosso, Christine E Horak, John Cogswell, Jason S Simon, Ashok K Gupta, Mario Sznol

**Affiliations:** 1 Memorial Sloan-Kettering Cancer Center, New York, NY, USA; 2 Duke University School of Medicine, Durham, NC, USA; 3 The Sidney Kimmel Comprehensive Cancer Center at Johns Hopkins, Baltimore, MD, USA; 4 Keck School of Medicine, University of Southern California, Los Angeles, CA, USA; 5 Bristol-Myers Squibb, Princeton, NJ, USA; 6 Yale University School of Medicine and Yale Cancer Center, New Haven, CT, USA

## Background

The fully human monoclonal antibodies nivolumab (Nivo) and ipilimumab (Ipi) block the interaction between the immune checkpoint receptors programmed death-1 (PD-1) and cytotoxic T lymphocyte antigen-4 (CTLA-4), respectively, and their cognate ligands, restoring antitumor immune response. Phase 1 studies of Nivo monotherapy (CA209-003; NCT00730639) or Nivo+Ipi combination therapy (CA209-004; NCT01024231) demonstrated durable clinical activity and objective response rates (ORRs) of 31% to 40%, respectively, in patients with advanced melanoma (MEL). Evaluation of PD-L1 expression using immunohistochemistry (IHC) suggested a correlation between pretreatment tumor PD-L1 expression and clinical response to Nivo monotherapy [1]. Identification of predictive markers of response would be valuable to guide effective use of Nivo and Ipi.

## Materials and methods

MEL patients received Nivo monotherapy (n=107) or Nivo+Ipi combination therapy (N=86; concurrent regimen: n=53; sequenced regimen: n=33). Tumor and tumor infiltrating lymphocyte (TIL) surface programmed death ligand 1 (PD-L1) expression in formalin-fixed, paraffin-embedded (FFPE) tumor tissue was evaluated by IHC with an automated assay (Dako) using the 28-8 Ab. Tumor PD-L1 positivity (PD-L1+) was defined as ≥5% cell membrane staining of any intensity; any expression on TILs was considered positive. Absolute lymphocyte counts (ALC) were measured in serial peripheral blood samples and lymphocyte subsets were evaluated using flow cytometry.

## Results

Tumor PD-L1-positive expression was observed in 45% and 37% of samples from the 003 and 004 studies, respectively. In 003, inclusion of any immune cell staining increased PD-L1 positivity to 92%. A numerically higher ORR was observed in MEL patients with PD-L1+ tumors with Nivo monotherapy or with sequential but not concurrent combination therapy. Neither study demonstrated an obvious change in ALC; however, phenotypic changes in T-cell subsets, including increases in the percentage of CD4 and CD8 expressing HLA-DR, ICOS and/or Ki67, were seen with combination therapy. In both studies, responses were observed irrespective of tumor PD-L1 or ALC status. In an exploratory analysis low pretreatment myeloid derived suppressor cells (MDSC) correlated with higher ORR with combination therapy (*P*< 0.05).

## Conclusions

PD-L1 positivity is associated with tumor response with Nivo monotherapy; however, some responses were observed independent of PD-L1 or ALC status. No correlation between response and PD-L1 or ALC status was seen with combination therapy. MDSC levels may correlate with response to combination therapy. Future phase 3 randomized studies will explore these markers and other phenotypic changes in immune cell populations that might predict activity of Nivo in patients with MEL and other advanced cancers.
